# A Rare Case of Schwannoma of the Right Greater Palatine Nerve in a 17-Year-Old Female Patient

**DOI:** 10.7759/cureus.56836

**Published:** 2024-03-24

**Authors:** Lyuben Stoev, Yanko G Yankov, Nikolay I Nikolaev, Martina Stoeva

**Affiliations:** 1 General and Clinical Pathology, Forensic Medicine and Deontology, Medical University of Varna, Varna, BGR; 2 Maxillofacial Surgery, University Hospital St. Marina, Varna, BGR; 3 General and Operative Surgery, Medical University of Varna, Varna, BGR

**Keywords:** nerve diseases, oral pathology, nerve sheath, hard palate plastic, greater palatine nerve, pathology, oral surgery, maxillofacial surgery, benign tumor, oral cavity

## Abstract

Schwannomas are not uncommon in the maxillofacial region; however, those with intraoral localization and, in particular, the hard palate are among the least frequently described. In the current case report, we present a 17-year-old girl with a histologically verified schwannoma of the hard palate on the right, originating from the right greater palatine nerve. In her case, despite the lysis of the palatine bone from the tumor compression, the disease is asymptomatic, causing only a weak sensation of local discomfort. The lesion was removed surgically under general anesthesia and the resulting defect of the palatal mucosa was compensated by plastic reconstruction with a lingual mucosal flap on a posterior feeding base. The recovery period was uneventful.

## Introduction

Schwannomas are benign tumors originating from Schwann cells, a type of glial cell responsible for producing myelin for the myelin sheaths of nerves [1,2]. They were first described by Virchow in 1908, who then gave them the name neurinomas, and in 1935, Stout called them neurilemomas [2]. Nowadays, some authors also call them peripheral fibroblastomas and peripheral gliomas [1]. Schwannomas are well-circumscribed, encapsulated tumors that can develop both intra- and extracranially. Between 25% and 40% of all schwannomas occur in the head and neck region, with only 1% of these having an intraoral location, primarily involving the mobile areas of the tongue, floor of the mouth, palate, vestibule, and lips [2,3]. In extremely rare cases, they can be intraosseous [2,3].

In this case report, in order to strengthen the clinical knowledge about schwannomas, we present a relatively rare case of this benign entity of the hard palate, arising from the myelin sheath of the right greater palatine nerve in a female teenager.

## Case presentation

We present a 17-year-old Caucasian female patient who was brought by her parents to a maxillofacial surgeon in January 2024 for an enlarging and uncomfortable lump on the right hard palate of approximately one year's duration. The lesion is asymptomatic at rest and causes no pain when compressed or during eating. The patient has no accompanying diseases, does not take medication, does not report allergies to food and medication. In 2009, at the age of three, tonsillectomy of the pharyngeal tonsil was performed in an ENT clinic under general anesthesia due to frequent and recurrent infections of the upper respiratory tract.

**Figure 1:**
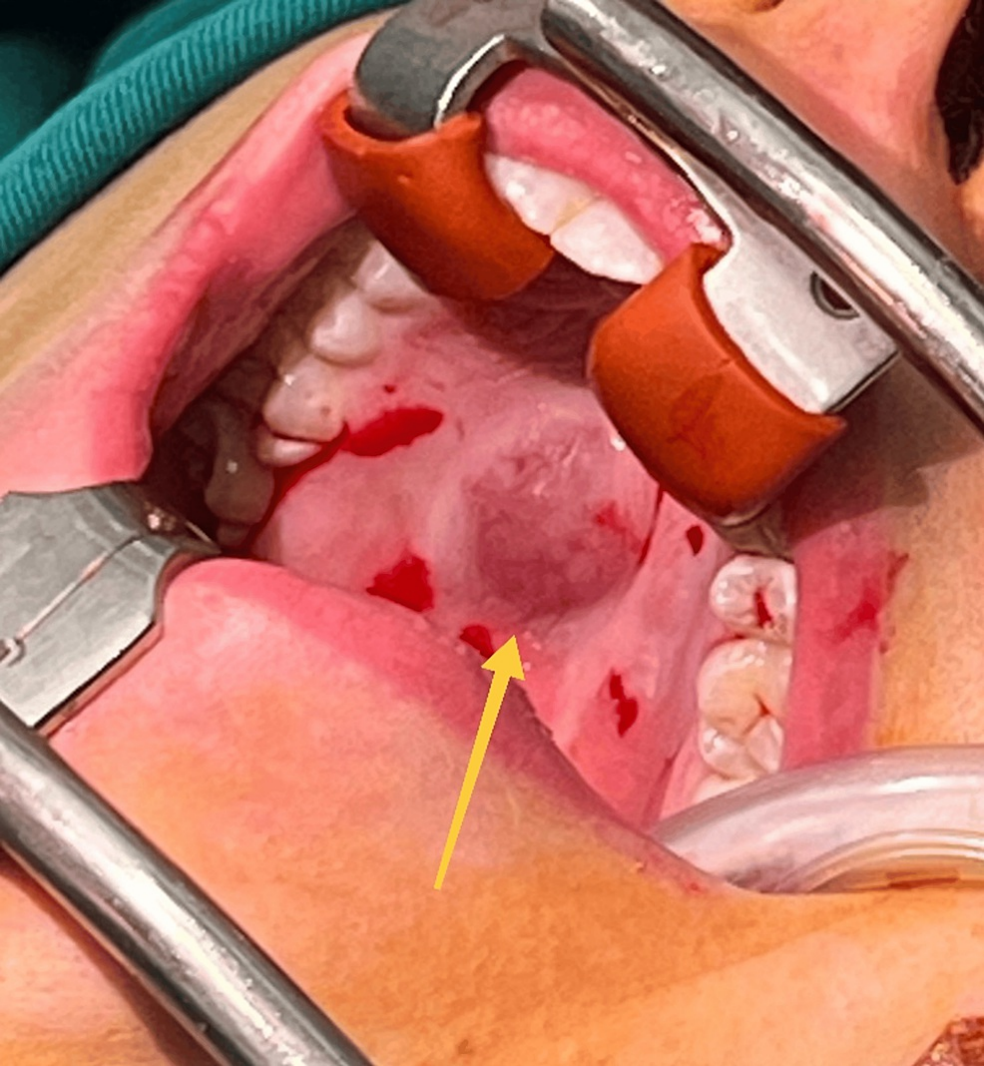
A photograph of the mass (yellow arrow) taken after intubation and the application of local anesthesia (the places around it where the punctures were made are visible) before the start of its surgical removal

Clinical examination by a maxillofacial surgeon revealed the presence of a painless, soft-elastic lump approximately 2.0 cm in diameter in the middle third of the hard palate right paramedian (adjacent to the midline and sagittal palatal suture). It was covered with a mucous membrane that is darker violet in color than the surrounding mucosa and has a smooth surface (Figure 1).

**Figure 2:**
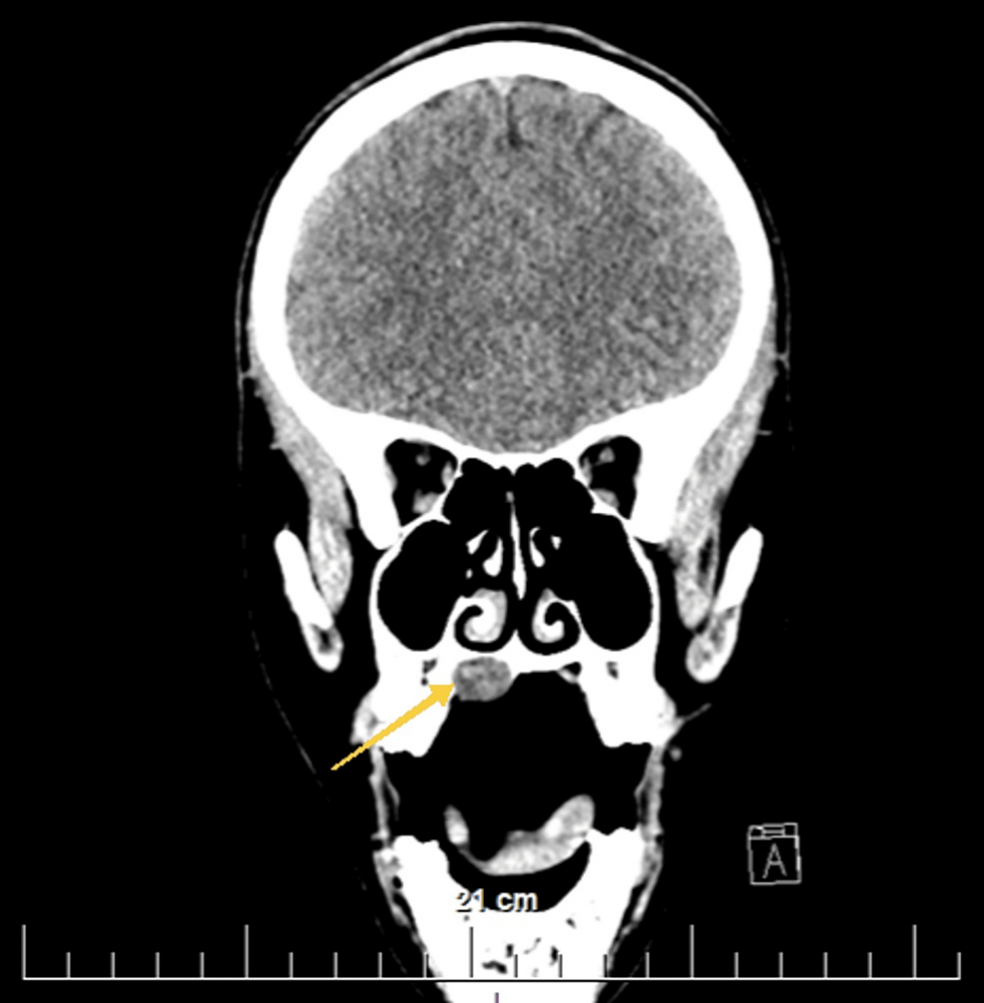
Transverse section of a native head CT. A yellow arrow indicates the location of the hard palate lesion on the right

**Figure 3:**
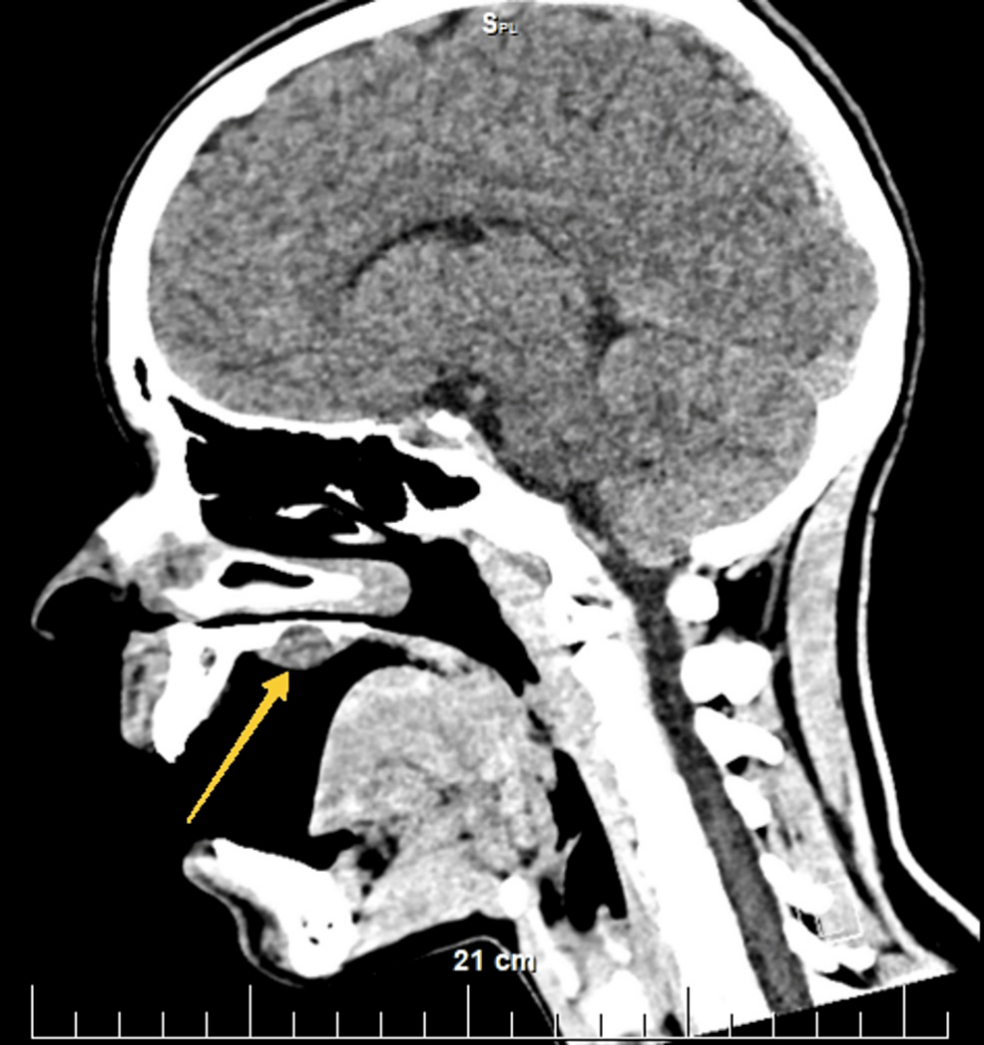
Sagittal section of a native head CT. A yellow arrow indicates the location of the hard palate lesion on the right. Above it, the thinning of the bony plate of the hard palate can be seen

**Figure 4:**
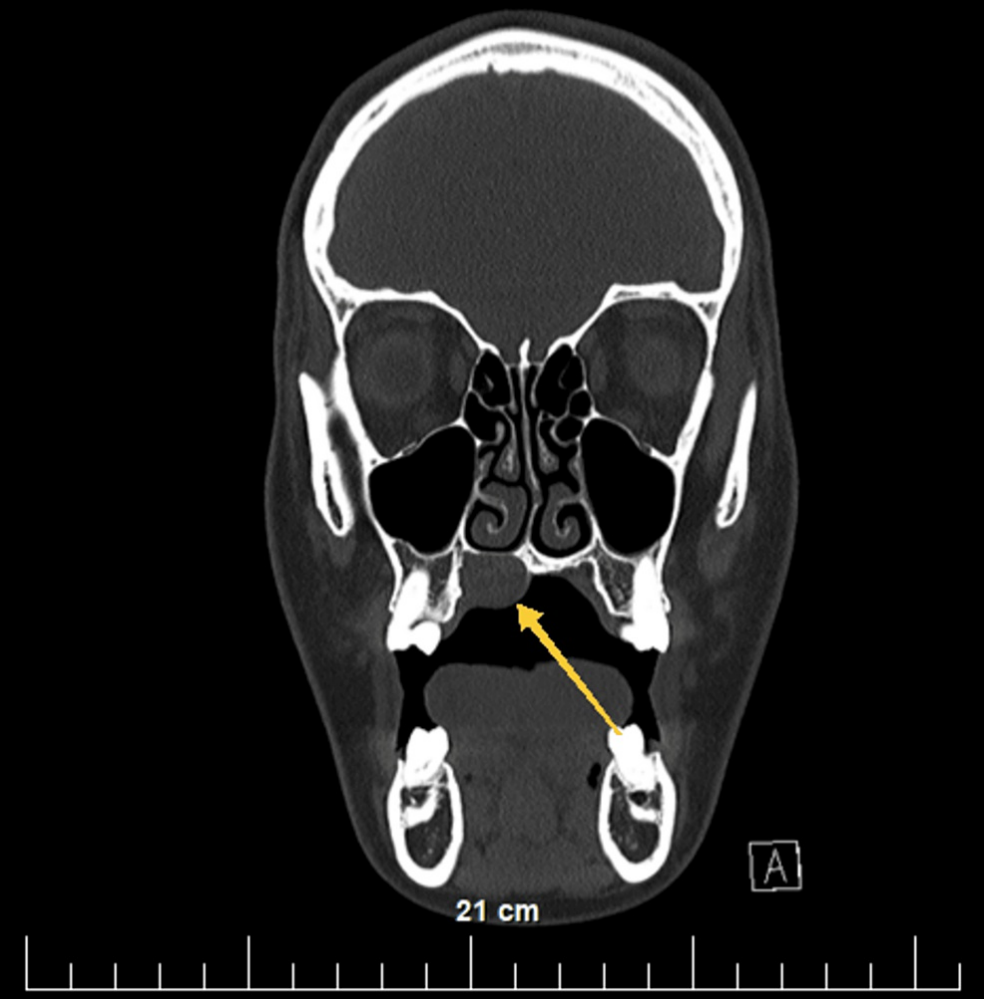
Transverse CT section of the head after intravenous administration of contrast material. A yellow arrow shows the location of the lesion on the right hard palate and the bony plate thinning

**Figure 5:**
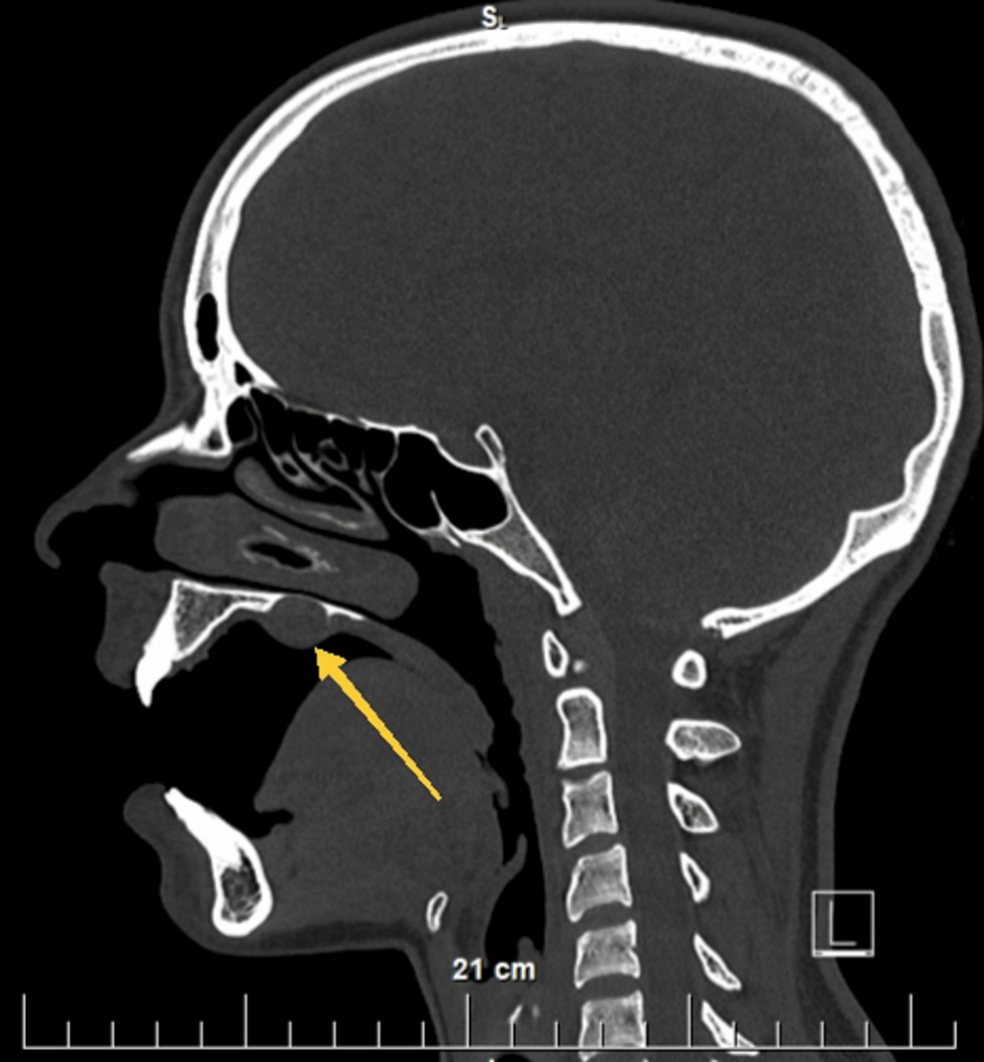
Sagittal CT section of the head after intravenous administration of contrast material. A yellow arrow shows the location of the lesion on the right hard palate and the bony plate thinning

Preoperatively, a complete blood count, biochemical indicators (glucose, urea, and creatine), coagulation status, urine, and urine sediment were examined, all without deviations from the reference limits for age and gender. Electrocardiography showed no abnormalities. CT of the head and neck was performed, native and after the intravenous administration of contrast material. The lesion showed a rounded shape; the thinning of the bony support of the hard palate above the formation is visualized when comparing it with the contralateral side (Figure 2, Figure 3, Figure 4, and Figure 5). No other deviations from the anatomical norm were found and there was no cervical lymphadenopathy. 

In terms of differential diagnosis, a retention cyst (mucocele) of a small salivary gland of the palatal mucosa was discussed and that assumption was dropped due to the appearance of the material after its excision.

**Figure 6:**
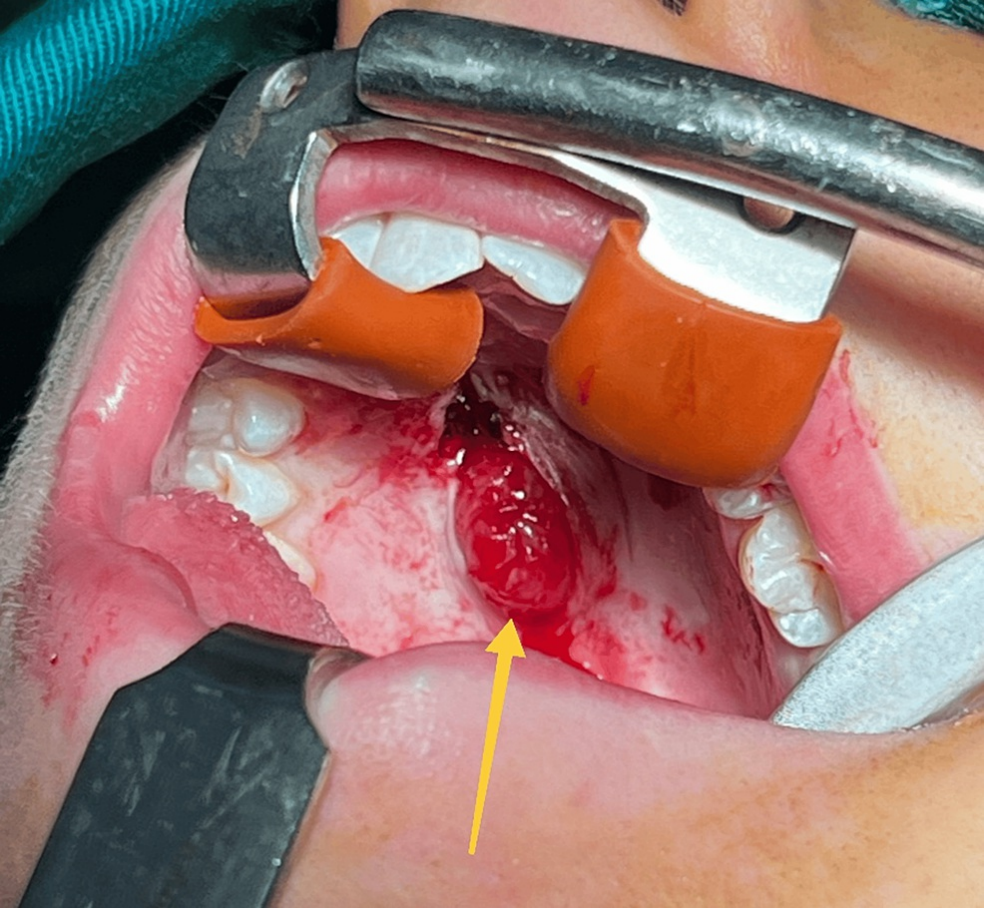
Intraoperative photograph. The lesion is marked with a yellow arrow

The surgical intervention was performed under general anesthesia with orotracheal intubation, after a thorough antiseptic of the operative field and after infiltration of local anesthetic lidocaine 2% with adrenaline in a ratio of 1:100000 for hemostasis. Through a linear incision of the mucosa above the formation in a sagittal direction, its capsule was reached (Figure 6) and the same was removed together with it within apparently healthy limits.

**Figure 7:**
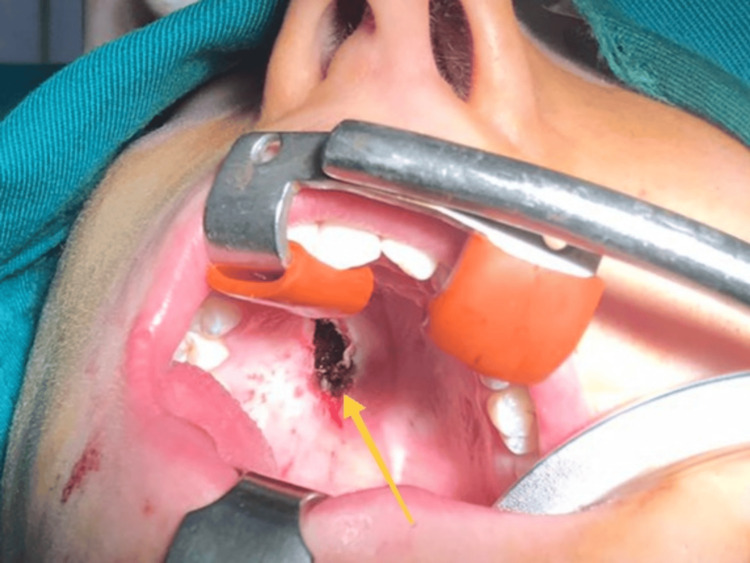
Intraoperative photo where the tumor bed is marked with a yellow arrow

**Figure 8:**
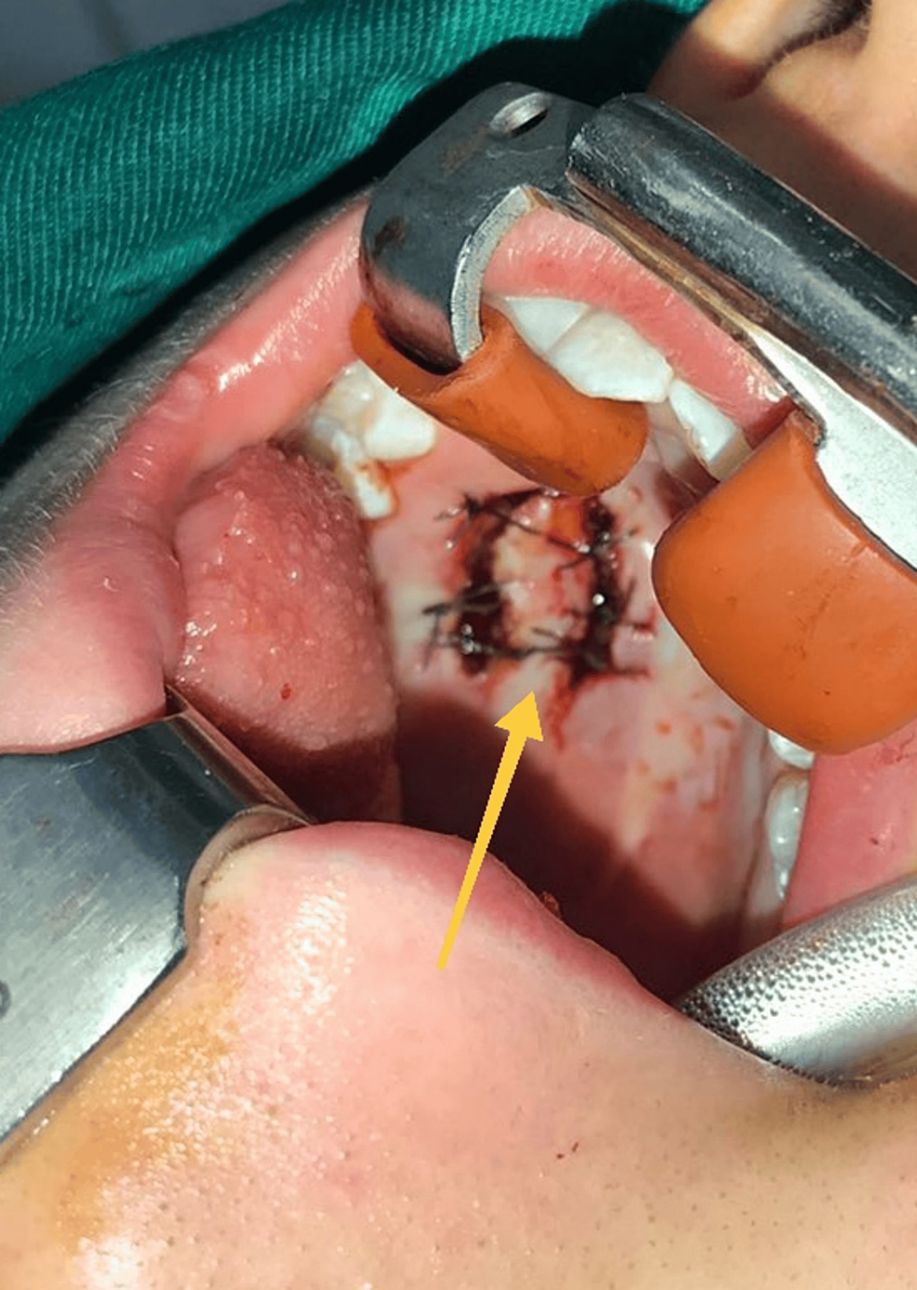
Intraoperative photograph showing the formed and sutured mucosal flap with a yellow arrow

The underlying bone plate was reached, the integrity of which was preserved. After lavage with saline, meticulous hemostasis was performed. To compensate for the existing soft tissue defect (Figure 7), a mucosal flap was formed on a posterior feeding base without interrupting the underlying periosteum, which was adapted and sutured with resorbable polyfilament (Figure 8).

**Figure 9:**
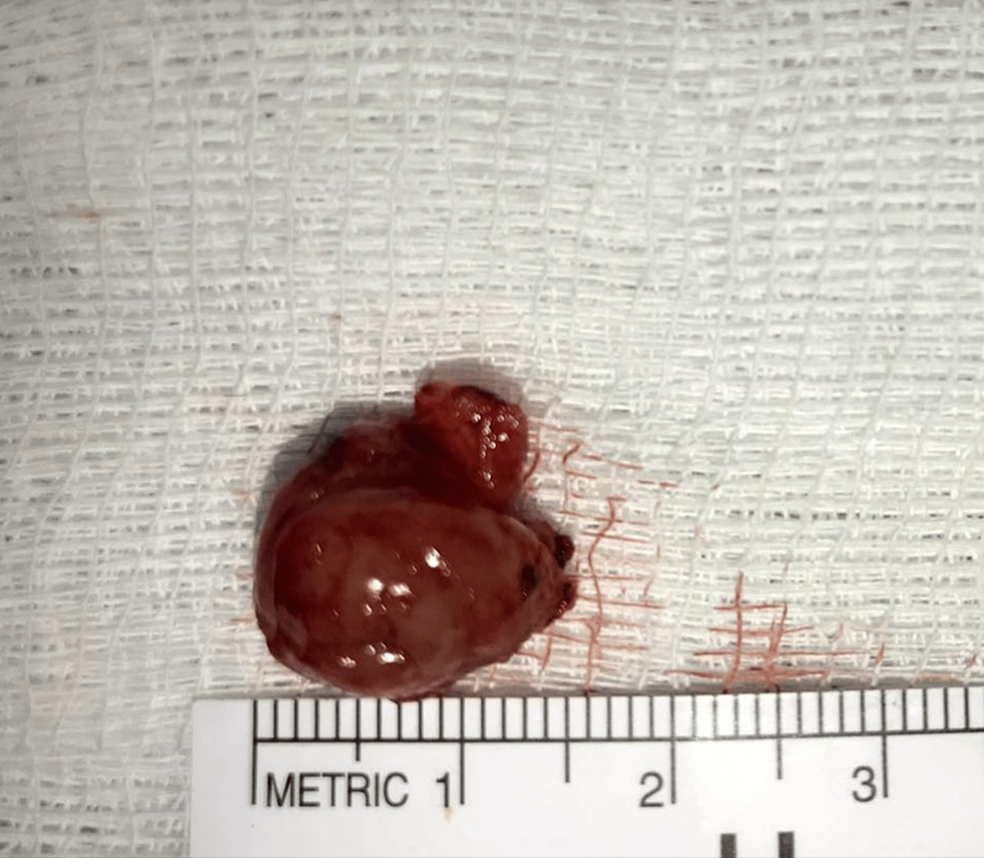
Photograph of the excised tumor material

The removed tumor was round in shape and with a smooth surface, measuring about 1.6 cm in diameter (Figure 9).

**Figure 10:**
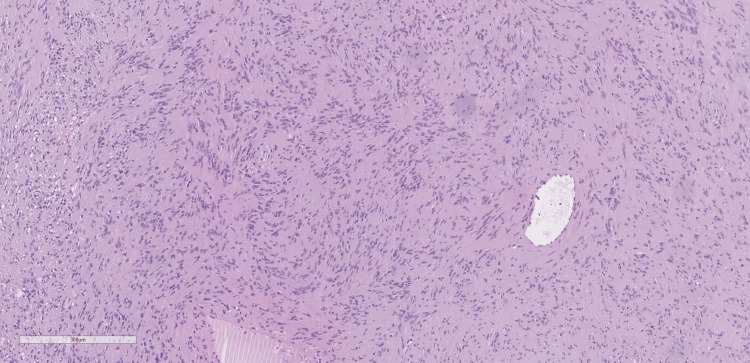
The lesion was predominantly composed of Antoni A cellular areas, H&E x10

**Figure 11:**
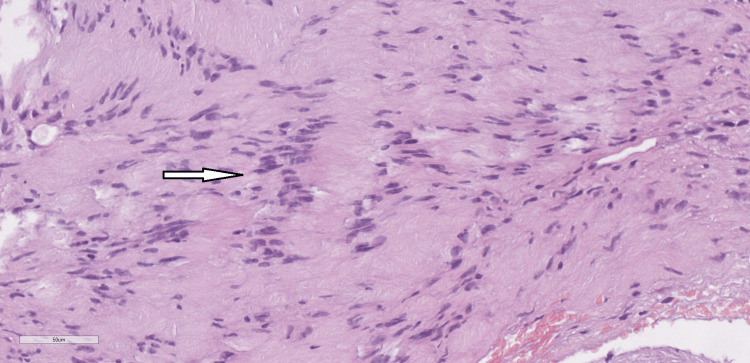
Verocay body (arrow), H&E x40

**Figure 12:**
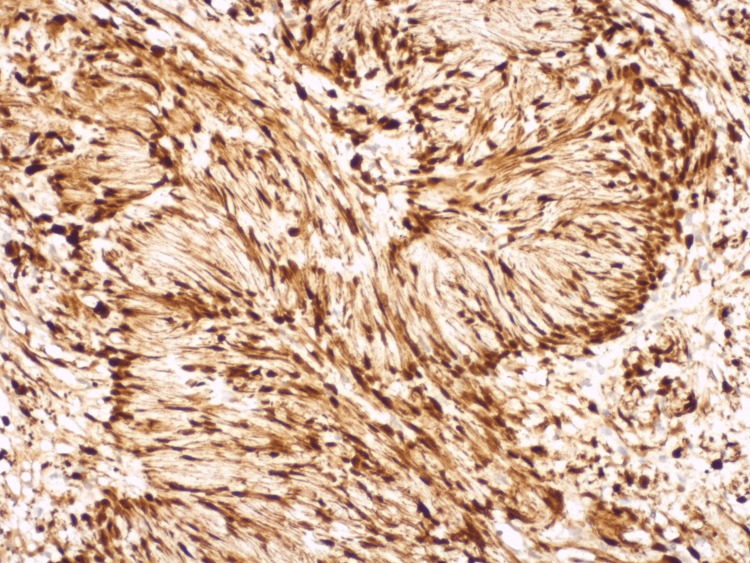
IHC staining for S100 protein shows diffuse and strong nuclear and cytoplasmic staining in the lesional cells, H&E x100

The histological report on the excised lesion revealed a schwannoma. Microscopically, the tumor was well-circumscribed but unencapsulated. The lesional cells were elongated and tapering, with monomorphic bland nuclei and ill-defined cytoplasm. Cellular Antoni A pattern of growth was uniformly dominating the histological picture, while the loose Antoni B pattern was barely present. Numerous Verocay bodies (palisaded nuclei around fibrillary processes) were found throughout the tumor. Large ectatic vessels were identified, but they showed no features of hyalinization (Figure 10 and Figure 11). Immunohistochemically, the tumor cells showed diffuse and strong expression of S100 protein (Figure 12).

Postoperatively, during the hospital stay of the patient, the antibiotic cefazolin was prescribed for preventive purposes, 2 g intravenously three times a day for two days. The patient was discharged on the second postoperative day in good general condition, with stable vital signs, without complaints and edema, and with a calm operative wound. She was prescribed antibacterial therapy at home, twice 0.5 g cefuroxim orally for seven days.

**Figure 13:**
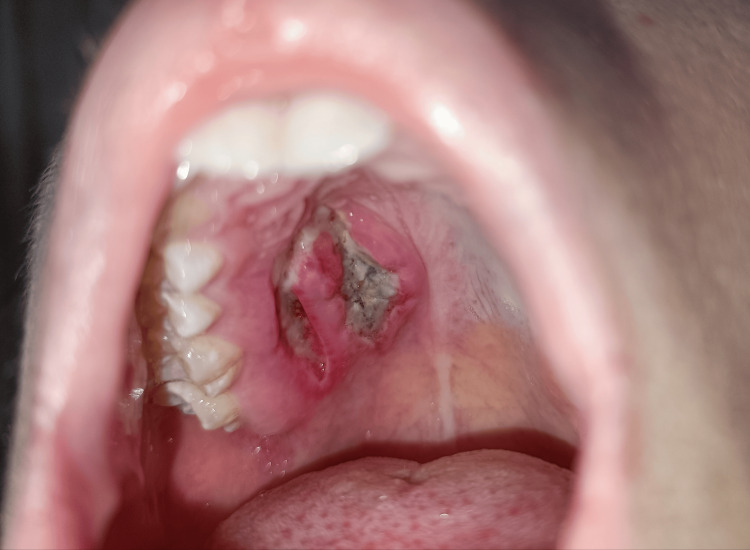
Photograph after suture removal on the 11th postoperative day

On the 11th postoperative day, the sutures were removed. The wound was painless, without edema, and with a normally shaped fibrin plaque along its edges (Figure 13).

## Discussion

The etiology of schwannomas is unknown, with chronic trauma and radiation exposure being the most common hypotheses [2,3]. Although usually solitary and sporadic lesions, they are often associated with certain genetic diseases, such as neurofibromatosis types I and II and schwannomatosis [1]. Schwannomas can occur at any time in life, with those with an extraoral location most common in adults with a peak between the second and fifth decades and those in the oral cavity between the second and third decades [4]. They show no clear gender predilection [2]. Schwannomas are common in the head and neck regions, but oral schwannomas are rarely reported. From the intraoral sites, the tongue is the most reported location of origin, while the hard palate is much more infrequent [5].

The main symptom of a schwannoma is a feeling of discomfort due to a newly appearing, slowly growing swelling [2]. The differential diagnosis includes all benign mesenchymal lesions of the oral cavity, tongue, and oropharynx - fibromas, leiomyomas, lipomas, and others [4]. Benign tumors like pleomorphic adenoma and low-grade malignancies of minor salivary gland origin like mucoepidermoid carcinoma often present as asymptomatic swellings and should be considered in this aspect [6]. Treatment of schwannomas requires surgical excision within healthy surgical margins, where it is essential to preserve the integrity of the nerve endings from which they arise [3]. Malignant transformation is exceptionally rare [3].

Histologically, schwannomas typically present as well-circumscribed lesions but encapsulation shows significant variability among cases. Incomplete capsular formation or total absence of a capsule is not an uncommon feature in oral sites [5]. The cellular composition is of spindle cells with elongated dark nuclei. Two patterns, compact cellular (Antoni A) and loose less cellular (Antoni B), are documented in variable proportions with Antoni A being dominant in most oral lesions. Palisaded Verocay bodies are another classical finding and are usually observed in oral schwannomas. Hyalinized vessels, although very characteristic of this histological type, are least frequently observed in the oral cavity among other head-and-neck locations [7]. On immunohistochemistry, schwannomas are diffusely positive for mature Schwann cell markers, including S100 and SOX10 [8]. The microscopic features of the current case fully match those described in the literature: good circumscription with a lack of well-defined capsule, Antoni A predominance with very scarce Antoni B areas, and complete absence of hyalinized vessels [7]. S100 was diffusely and strongly positive throughout the whole lesion.

Most often, schwannomas originate from the glossopharyngeal, vagus, accessory, and hypoglossal nerve [9]. The general sensory and parasympathetic autonomic innervation of the hard palate is carried out by branches of the pterygopalatine ganglion [10,11]. The anterior portion of the hard palate distal to the third teeth is innervated by the incisive nerve. It is a continuation of the longest branch, taking its origin from the pterygopalatine ganglion, one of the nasal branches and is called the nasopalatine nerve. The latter passes through the nasal cavity and merges with the nerve of the same name on the opposite side, forming the so-called incisive nerve appearing in the front of the hard palate at the incisive papilla [10]. The distal palatal areas backward from the fourth teeth receive innervation from both greater palatine nerves, also branches of the pterygopalatine ganglion, passing through the large palatine canal and exiting on the hard palate through the two greater palatine foramen [11]. The sagittal projection of the schwannoma excised by us on the hard palate in its area between the fourth and sixth teeth, a section that is innervated by the right greater palatine nerve, shows that it originates from the myelin sheath of this particular nerve.

## Conclusions

Hard palate schwannomas are uncommon benign tumors of Schwann cell origin, morphologically very similar to their soft tissue counterparts but usually lacking encapsulation and exhibiting predominantly the cellular Antoni A pattern of growth. The histological characteristics of the tumor presented in the current case report correspond well with the features of mucosal schwannomas described in the literature. Clinically such lesions may mimic other benign tumors of the oral cavity or low-grade malignancies. The definitive distinction can be achieved only through histological examination and immunohistochemical staining.
